# Prognostic role of follicular fluid tumor necrosis factor alpha in the risk of early ovarian hyperstimulation syndrome

**DOI:** 10.1186/s12884-020-03379-9

**Published:** 2020-11-12

**Authors:** Miaad Jabbar Alhilali, Abbas Parham, Armin Attaranzadeh, Malihe Amirian, Mohammad Azizzadeh

**Affiliations:** 1 Department of Physiology, College of Veterinary Medicine, AlQadisiyiah University, Diwanyiah, Iraq; 2 Division of Physiology, Department of Basic Sciences, Faculty of Veterinary Medicine, Ferdowsi University of Mashhad, Mashhad, Iran; 3 Stem Cell Biology and Regenerative Medicine Research Group, Institute of Biotechnology, Ferdowsi University of Mashhad, Mashhad, Iran; 4 Milad Center for Infertility Treatment, Mashhad, Iran; 5 Mashhad University of Medical Sciences, Mashhad, Iran; 6 Department of Clinical Sciences, Faculty of Veterinary Medicine, Ferdowsi University of Mashhad, Mashhad, Iran

**Keywords:** Tumor necrosis factor- alpha, Ovarian hyperstimulation syndrome, Larger follicles count, Cytokines, Intracytoplasmic sperm injection

## Abstract

**Background:**

Ovarian hyperstimulation syndrome (OHSS) is an iatrogenic condition characterized by capillary hyperpermeability which can be predicted by preovulatory ovarian responses such as number of follicles. A variety of cytokines are thought to be involved in pathophysiology of this syndrome.

**Methods:**

A prospective cohort study invloving sixty intracytoplasmic sperm injection (ICSI) patients. On the day of hCG injection, we explored the threshold of larger follicles ≥11 mm diameter with a count of ≥18 follicles for the high-risk moderate-to-severe OHSS and 13–18 follicles for the low-risk moderate-to-severe OHSS. Whereas larger follicles count of less than 13 were classified as normoresponders. Pooled follicular fluid (FF) samples of each patient were collected on the day of oocyte retrieval. Magnetic multiplex immunoassay was explored to measure the concentrations of some intrafollicular cytokines including: GM-CSF, INF-γ, TNF-α, IL-10, CXCL8/IL-8, IL-6, IL-5, IL-4, IL-2, and IL-1β. All sixty patients underwent controlled ovarian hyperstimulation (COH) with either GnRH agonist or antagonist protocols.

**Results:**

Intrafollicular TNF-α concentration was significantly different (p < 0.05) in the high-risk moderate-to-severe OHSS patients compared to low-risk moderate-to-severe OHSS patients and normoresponders. TNF-α in FF had a negative correlation with the chance of high-risk moderate-to-severe OHSS. The differences in the risk of OHSS between patients who received GnRH agonist or antagonist were not significant (p > 0.05).

**Conclusions:**

In accordance to the negative correlation of TNF-α and high risk of early OHSS, we did not expect TNF-α to play a role in increasing vascular permeability in ovarian tissues. In addition, the risk of early moderate-to-severe OHSS was not affected by different GnRH superovulation protocols.

## Background

Ovarian hyperstimulation syndrome (OHSS) is an iatrogenic condition characterized by capillary hyperpermeability [1]. Early OHSS occurs about 1–9 days after human chorionic gonadotropin (hCG) treatment and is related to gonadotropin administration [2]. It can be predicted by in-time preovulatory ovarian response to institute preventative methods [3]. Whereas the late form of OHSS occurs about (10 days) after hCG treatment as a result of placental hCG [2]. This form of OHSS does not correlate intensely with preovulatory ovarian response, making it problematic to recognize cycles where it is likely to occur [4]. Although the definitive physiologic mechanism of OHSS is not yet identified, there are well-known risk factors that must be considered during the administration of medications for infertility treatment [1]. It seems that a combination of non-immune (e.g. hCG, renin-angiotensin system and luteinizing hormone (LH)) and immune (e.g. cytokines) mechanisms may allow a profound understanding of this syndrome. The relationship found between plasma cytokine activities and the severity of this syndrome proposes that plasma cytokines may be involved in the pathogenesis of OHSS and may act as a means of checking the syndrome [5].

A wide spectrum of vasoactive cytokines and growth factors are thought to be concerned in the pathophysiology of OHSS including vascular endothelial growth factor (VEGF), tumor necrosis factor- alpha (TNF-α), interleukin (IL)-10, IL-8, IL-6, and IL-2. The more important well-studied vasoactive cytokine is VEGF, which may be implicated in the pathogenesis of OHSS. It regulates endometrial vascularization, vascular permeability, supports trophoblast invasion and blastocyst implantation [6].

TNF-α is produced from corpus luteum locally and has a role in angiogenic activity modulation throughout the luteal phase [7]. TNF-α is one of the vital cytokines in growth and selection of antral follicle; the local dose of TNF-α may determine the follicular response [8]. A suggested model for participation of the immune system in the pathophysiology of the syndrome proposed that hCG, LH, and other factors stimulate ovarian macrophages and granulosa cells to produce TNF-α and other cytokines, such as IL-2. The secreted TNF-α stimulates lymphocytes and natural killer cells resulting in activation of endometrium to increase vascular permeability and appearance of the clinical manifestations of OHSS [4]. Orvieto et al. (2014) proposed that women at risk to develop the syndrome have hereditary paradoxical immune response to hCG, with IL-2 instead of suppressor of cytokine signaling (SOCS-1) domination, resulting in systemic inflammatory response with the consequent OHSS/VLS (vascular leak syndrome) [9].

The previous studies were contradictory regarding to the protocols in reducing OHSS incidence. According to the usage of a gonadotropin-releasing hormone (GnRH) agonist versus antagonist analogue in ovarian stimulation, GnRH analogue protocols are categorized as GnRH antagonist or agonist protocols [10]. Some studies proposed the ability of GnRH antagonist or hCG withholding (cycle cancellation) in preventing OHSS incidence, while other studies completely controverted with this concept and their results [11]. They revealed that using long agonist protocol in young normo-gonadotropic women reduced occurrence of severe and moderate OHSS when this protocol is explored for ovarian superovulation [12].

Nastri et al., (2015) in their two large comprehensive studies suggested two groups of parameters before and during COH as the best parameters for predicting OHSS and high response. The first category of parameters before COH include basal serum follicle stimulating hormone, antral follicle count, and anti-M¨ullerian hormone. The second category of parameters during COH for successful prediction of OHSS are levels of E_2_, medium/large follicle count on the day of hCG injection, and the number of oocytes following the follicle aspiration [13]. Follicles number on this day can differentiate the women who are at risk of developing OHSS, while E_2_ concentration is less reliable for the prediction of a syndrome [14]. Therefore, we selected larger (medium/large) follicles count on the day of hCG injection in this study to classify early moderate-to-severe OHSS in our ICSI patients for investigating the possible correlation between some FF cytokines and the odds of OHSS in ICSI patients. Furthermore, the possible impact of different ovarian hyperstimulation protocols in the occurrence of this potentially life-threating syndrome was investigated.

## Methods

### Experimental models

The present study was part of a larger research project regarding the association of some folicullar fluid cytokines with intracytoplasmic sperm injection outcome (15). Based on the inclusion criteria, from the 80 patients of original project, sixty couples suffering from female infertility who were undergoing assisted reproduction treatment using ICSI in Milad Infertility Center, Mashhad University of Medical Sciences, Iran entered the study. The inclusion criteria were age of the female partner (20–38 years), lack of endometriosis, obesity, smoking, diabetes, or hypertension. They all underwent COH with GnRH protocols of ICSI. The sixty enrolled patients included (41/60) OHSS patients and (19/60) age-matched normoresponders. Thirty-three of patients (55%) were undergoing their first ICSI attempt and twenty-seven patients (45%) had two or more IVF/ICSI attempts. Furthermore, other case history information such as infertility duration, infertility type (primary/ secondary), history of previous OHSS, and endometrial thickness were recorded.

The previously described ovarian superovulation protocols by Alhilali et al. (2019) were used in this study [15]. Briefly, ovarian superovulation was performed by daily administration of recombinant FSH (Gonal-F; “Merck” Serono, Germany) with agonist long beginning at the mid-luteal phase (day 21) of the previous cycle and continued to the day of hCG injection, while the antagonist protocol was achieved using (Cetrotide; Merk Serono, Germany) daily administration at the mid-follicular phase after the size of follicles reached 12 mm at least. In all patients, hCG (Pregnyl; IBSA, the Netherlands) was explored to stimulate the final oocyte maturation. Follicles were monitored regularly, their number and size were recorded using ultrasonography. Ultrasound guided, transvaginal aspiration of ovarian follicles was done about 36 h following hCG intramuscular injection to pick up oocytes.

Pooled follicular fluid samples were collected from each patient, centrifuged and stored at − 80 °C until they were analyzed. Concentrations of cytokines/chemokine were measured by Flow Cytometry with Luminex Platform Magnetic Luminex. Human premixed multi-anaylte kit (R&D System Inc. Kit code: LXSAMH-10) was purchased from Bio-Rad laboratories, Italy. All the analyses were done in Lapospace laboratories, Milan, Italy, by Luminex instrument (Bio-Rad) (Luminex Map Technology, Milan, Italy). Samples were examined using Bio-Plex Manger software V 6.0. The procedure for evaluation of multiplex cytokines was performed according to the manufacturer’s instructions to estimate the following cytokines: GM-CSF, INF-γ, TNF-α, IL-10, CXCL8/IL-8, IL-6, IL-5, IL-4, IL-2, and IL-1β. Values of the standard curve are compared with the values provided by the manufacturer of the kits, which must exceed a percentage variance coefficient (CV %). All of the above parameters are applied on at least the 90% of the standard curve value. Follicular fluid cytokines assay techniques as well as follicle and oocyte quality were previously explained in details [15].

The classification of patients at risk of OHSS was achieved according to Papanikolaou et al. (2006). They recommended that increased medium/large follicles count ≥13 follicles ≥11 mm in diameter was the threshold of OHSS risk, and that threshold of ≥18 follicles ≥11 mm can predict more than 83% of severe cases. In addition, the likelihood for developing early OHSS might reach zero when follicles do not exceed 13 count [14]. The results of the latter study was confirmed by the study of Griesinger et al. (2016) where they proposed that on the day of hCG, the optimal threshold of ≥19 follicles ≥11 mm diameter was correlated with an increased risk for severe and moderate-to-severe OHSS. In addition, the number of follicles on this day is a better indicator compared with serum E_2_ levels in predicting OHSS [16]. Therefore, we explored the threshold of larger (medium/large) follicles count ≥18 follicles ≥11 mm in diameter on the day of hCG injection for high-risk moderate-to-severe OHSS group and 13–18 follicles for low-risk moderate-to-severe OHSS group. The other patients with larger follicle count of less than 13 were classified as normoresponders.

### Statistical analysis

Comparisons of ICSI outcomes among the three groups of the study was performed by using Kruskal-Wallis test followed by Mann-Whitney test with the Bonferroni correction for pairwise comparison. To select those explanatory variables that best explained the chance of OHSS, ordinal logistic regression was used. At the first step, explanatory variables including: superovulation protocol, previous IVF/ICSI attempts, and infertility type were entered in the model as categorical variables. In addition, age, infertility duration, and FF cytokines concentration were entered in the model as quantitative variables. Backward stepwise approach was explored to select those explanatory variables that best explained the chance of OHSS. The significance of each explanatory variable in the model was tested by Wald test. The insignificant explanatory variables were removed from the model one at a time, beginning with the least significant, until the estimated regression coefficients for all retained variables were significant at an alpha level of < 0.05.

## Results

The demographics of patients enrolled in this study are presented in Table 1. The sensitivity of cytokines detection (LLOQ and ULOQ), median (minimum- maximum) values of the intrafollicular cytokines in normoresponders and the two groups of OHSS, low-risk and high-risk moderate-to-severe OHSS groups are presented in Table 2. Embryo transfer was performed in 75% (45/60) and canceled in 25% (15/60) of patients. Cancellation of embryo transfer in those 15 patients was either due to the large number of oocytes retrieved (≥ 25 oocytes), history of OHSS, or developed clinical signs of OHSS.

**Table 1:** Patient’s demographics and cycle characteristics. Qualitative Variables were presented as frequency (%). Quantitative variables were presented as mean ± standard deviation

	Normoresponders *n* = 19	Low-risk OHSS *n* = 13	High-risk OHSS *n* = 28
Age (year)	32.11 ± 5.51	29.0 ± 3.70	29.46 ± 3.94
Infertility duration (year)	4.89 ± 4.22	5.96 ± 3.61	6.04 ± 3.42
*Superovulation protocol*			
Agonist	7 (11.7%)	3 (5%)	15 (25%)
Antagonist	12 (20%)	10 (16.7%)	13 (21.6%)
*Previous IVF/ICSI attempts*			
No	9 (15%)	7(11.7%)	17 (28.3%)
Yes	10 (16.7%)	6 (10%)	11 (18.3%)

**Table 2:** Median (minimum-maximum) intrafollicular cytokine concentrations, LLOQ, ULOQ (pg/ml) of normoresponders, and moderate-to-severe OHSS groups of the study

Cytokine (pg/ml)	Normoresponders ***n*** = 19	Low-risk OHSS ***n*** = 13	High-risk OHSS ***n*** = 28	LLOQ (pg/ml)	ULOQ (pg/ml)
**GM-CSF**	12 (10–13)	11.5 (10–12.5)	11.5 (10–60)	11.9	2881.1
**IL-1β**	13 (11.5–14)	13 (12–13.8)	13 (12–15.3)	16.2	3961
**IL-6**	34 (18.5–186.3)	34 (22–435.5)	28 (18–461)	4.5	1103.3
**IL-2**	33 (16.5–37.8)	30 (23.5–37.5)	31.15 (22.8–36)	28.8	7439.4
**IFN-γ**	18 (16.8–19.5)	18 (16–18.8)	17.5 (15.5–22)	49.2	11,949.1
**TNF-α**	12 (11–13)	12 (10.5–13)	11 (10.5–12.5)*	8.9	2161.5
**CXCL8/IL-8**	760.8 (309–1863.8)	697.8 (206.3–1796.8)	650.3 (207–1339.5)	4.4	1060.6
**IL-10**	43.8 (34.8–60)	38.0 (33.5–69.0)	47.65 (32.5–67.5)	7.3	882.9
**IL-4**	16 (13.8–17.8)	15.8 (14.5–17.5)	15.5 (14–17.3)	15.4	3750
**IL-5**	10.5 (9.5–11.5)	10.0 (9–11)	10.15 (9–12)	6.3	1539.2

* Differences were statistically significant (*p* <0.05). Values are presented as median (minimum- maximum) in the three groups of the study; *LLOQ* Lower limit of qualification, *ULOQ* Upper limit of qualification, *GM- CSF* Granulocyte macrophage-colony stimulating factor, *IL* interleukin, *INF-γ* interferon gamma, *TNF-α* tumor necrosis factor-alpha

**Fig. 1:**
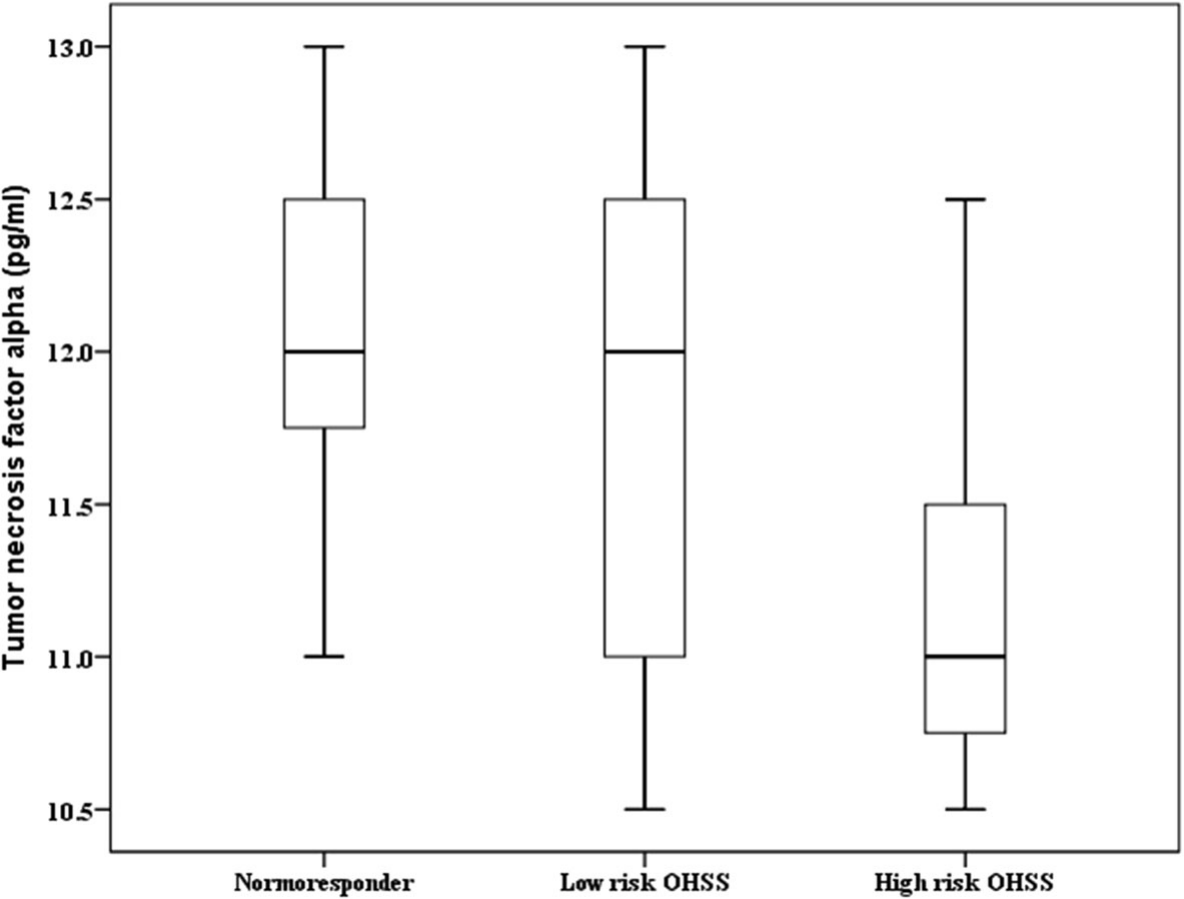
Box blot showing the negative correlation between intrafollicular TNF-α concentration (pg/mL) and OHSS in the three groups of the study. OHSS; ovarian hyperstimulation syndrome

TNF-α was correlated significantly and inversely with the severity of OHSS. An increase of each picogram of TNF-α in follicular fluid decreased the chance of moderate-to-severe OHSS approximately by one third (*p* = 0.001; OR = 0.27). Other nine measured cytokines in FF including GM-CSF, IFN-γ, IL-10, IL-6, IL-5, IL-4, IL-2, IL-1β, and CXCL8/IL-8 had no significant correlation with the chance of OHSS. The values of TNF-α in the three groups of the study were presented in Fig. 1.

Twenty-five patients (41.7%) received a daily dose of agonist long recombinant FSH (Gonal-F; Merk Serono, Germany). The other 35 patients (58.3%) received daily administration of antagonist drug (Cetrotide; Merk Serono, Germany). There were no significant differences between normoresponders and the two groups of OHSS respective to their superovulation protocols (*p* = 0.251).

Age was inversely correlated with odds of OHSS, but the differences between normoresponders and the two groups of OHSS were insignificant (*p* = 0.853). Infertility duration and number of previous IVF/ICSI attempts did not associate significantly with the odds of OHSS, the significance for these parameters was (*p* = 0.624), and (*p* = 0.681), respectively.

The number of oocytes was significantly different between normoresponders and OHSS groups. Number of oocytes was significantly higher in both OHSS groups than normoresponders (*p* = 0.001), whereas the number of oocytes within the two groups of OHSS did not differ significantly (*p* = 0.151).

## Discussion

The current study revealed a significant negative correlation between intrafollicular TNF-α concentration on the day of oocyte retrieval and the risk of moderate-to-severe OHSS in our ICSI patients. In addition, the risk of early moderate-to-severe OHSS was not affected by different GnRH superovulation protocols. The present study was part of a larger research project (15) and we found 19 normoresponder and 41 OHSS (13 low-risk and 28 high-risk) patients during our investigation.

We explored the number of larger (medium/large) follicles on the day of hCG injection in the classification of early OHSS. The number of larger follicles stimulated during COH and assessed by ultrasound reflects the degree of ovarian response. The greater the triggering on the time of hCG stimulation, the greater the risk of OHSS [13]. Agrawal et al. (1999) explored the number of follicles for prediction of the risk of moderate-to-severe OHSS with a sensitivity of 100% and specificity of 56% [17]. Lee et al. (2008) concluded that follicles count ≥11 mm diameter on hCG day correlated with the ovarian reserve and response to COH [18]. The retrospective study of Ashrafi et al. (2015) revealed that on hCG day follicles count ≥17 ≥ 12 mm diameter may be a valuable indicator for discriminating women at high risk for moderate-to-severe early OHSS [2]. Overall, we can conceive the importance of larger follicles count ≥11 mm on hCG day in the prediction of patients at risk of OHSS.

Some previous studies found a significant increase in TNF-α in moderate and severe OHSS patients [4, 19, 20], while others revealed no significant correlation between OHSS incidence and TNF-α concentrations in FF [21]. In the preovulatory stage, TNF-α is one of the essential factors, however, the definite role and effect of TNF-α in the ovary are not entirely understood [22]. TNF-α is a T helper-1 (Th-1) multifunctional hormone-like polypeptide, which has a wide range of biological roles. In ovary, macrophages are the major source of TNF-α, but TNF-α is also produced from oocytes, corpora lutea, and the cells of theca and granulosa. This cytokine acts by binding to two types of receptors: TNF-α –R1 is mostly responsible for the transduction of death signal, while TNF-α –R2 is mainly concerned in cell proliferation. It seems that in ovary, TNF-α can initiate either apoptosis or proliferation according to the stage of follicular progress and the type of the cell [23].

Cytokines are well known to regulate the paracellular permeability in epithelial and endothelial cells through mediation of tight junctions. Previous studies have found discrepant effects of TNF-α on paracellular permeability. While TNF-α increases permeability in human umbilical vascular endothelial cells (HUVECs), human colonic adenocarcinoma (Caco-2), and bovine pulmonary artery endothelial cell (BPAEC), this cytokine decreases the permeability in uterine epithelial cells (UEC) and porcine renal epithelial cells (LLC-PK1). There is no clear reason for this discrepancy between the functions of cytokines on the endothelial and epithelial systems. However, it may refer to cell-specific mechanisms for tight junction remodeling [24]. Therefore, we did not expect TNF-α to have a role in increasing vascular permeability in ovarian tissue. Thereby, the pathophysiology of OHSS may be attributed to the effect of other cytokines other than TNF-α.

In our study, TNF-α levels significantly decreased in FF of high-risk moderate-to-severe OHSS patients than in normoresponders and low-risk OHSS group. We speculate that the lower concentrations of TNF-α in FF of moderate-to-severe OHSS group was due to the injection of hCG. Studies in animal models established the immunomodulatory role of hCG through downregulation of target cells involving Th-1 cells and suppressing their production [25]. In current study, we used hCG to trigger final maturation in all patients. The hCG may stimulate OHSS occurrences due to the triggering effect of hCG on granulosa lutein cells in the corpus luteum. Consequently, rises in the endothelial permeability results in the appearance of clinical manifestations of OHSS [26]. Drakopoulos et al. (2016) suggested that the usage of hCG to trigger final maturation of oocytes instead of GnRH agonist will increase the risk of OHSS, especially in high ovarian response patients [27].

FF TNF-α concentration in our study was lower in severe OHSS group who had a higher oocyte number than in normoresponders. During folliculogenesis, TNF-α has inhibitory actions on the dominant follicle selection and Graafian follicle stages [23]. It appears that reducing the number of released oocytes and stimulating granulosa cell death of un-ruptured follicles enhances follicle atresia. These effects are mediated by TNF-α through autophagy and apoptosis [22].

Many studies detected the levels of other cytokines in serum, FF, peritoneal fluid, and peripheral blood mononuclear cells (PMNCs) as an early predictor for severe OHSS [9, 20, 28, 29]. For example, peritoneal fluid IL-10 is involved in pathogenicity of severe OHSS [30] and significantly increased in FF of severe OHSS patients [20]. Another study revealed the elevated concentration of other cytokines including IL-8, IL-6, and TNF-α in peritoneal fluid, IL-1β and IL-6 from serum of patients with severe OHSS [28]. These findings suggested that these cytokines might be implicated in mediating the increased capillary permeability in OHSS. However, the concentrations of IL-1β, IL-10, IL-8, and IL-6 did not differ significantly from FF of our OHSS patients and those who did not have OHSS.

The main purpose of conventional superovulation protocols in assisted reproductive treatment is to increase the number of oocytes retrieved. Nevertheless, these protocols correlated with amplified risk of OHSS, especially in those patients producing oocyte count exceeding 15 [31]. It is believed that substituting hCG by recombinant LH or GnRH agonist, antagonist protocols, and dopamine agonists decrease the incidence of OHSS. The use of antagonist has the benefit of decreasing the rate of incidence of the syndrome in high-risk patients [13, 29]. Usage of antagonist protocol combined with the GnRH agonist for triggering final oocyte maturation significantly decreases OHSS, especially in high-risk patients [27]. However, we did not find significant differences between the use of these two types of protocols and the incidence of OHSS in our patients.

According to a large body of previous investigations, the majority of patients with OHSS are younger than those who do not [11, 16, 32, 33]. Younger age is considered as one of main risk factors for developing OHSS [11] since OHSS depends on the ovarian reserve of the patient; the ovaries of a younger patient has a larger amount of gonadotropin receptors or a higher follicles count which are capable of responding to gonadotropins [32]. In our ICSI patients, although age was inversely correlated with OHSS incidence, it was not significant.

## Conclusion

In conclusion**,** our results revealed a significant negative correlation between intrafollicular TNF-α concentration on the day of oocyte retrieval and the risk of moderate-to-severe early OHSS. According to this negative correlation, we did not expect TNF-α to have a role in increasing vascular permeability in ovarian tissues. Thereby, the pathophysiology of OHSS may be attributed to the effect of cytokines other than TNF-α. In addition, the moderate-to-severe risk did not affect the different GnRH superovulation protocols. Furthermore, age did not significantly correlate with different forms of OHSS severity.

Overall, it can be concluded that the evaluation of intrafollicular cytokines, TNF-α in this study, has a prognostic role in the prediction of patients at risk of developing early moderate-to severe OHSS in ICSI patients.

## Data Availability

The data sets used and/or analyzed during the current study are available from the corresponding author on reasonable request.

## Abbreviations

OHSS: Ovarian hyperstimulation syndrome
PCOS: Polycystic ovary syndrome
ICSI: Intracytoplasmic sperm injection
COH: Controlled ovarian hyperstimulation cycles
FF: Follicular Fluid
hCG: Human chorionic gonadotropin
TNF-α: Tumor necrosis Factor- α
GM-CSF: Granulocyte macrophage- colony stimulating factor
INF-γ: Interferon- γ
IL: Interleukin
VEGF: Vascular endothelial growth factor
